# Characterization of a New Toti-like Virus in Sea Bass, *Dicentrarchus labrax*

**DOI:** 10.3390/v15122423

**Published:** 2023-12-13

**Authors:** Lénaïg Louboutin, Joëlle Cabon, Véronique Beven, Edouard Hirchaud, Yannick Blanchard, Thierry Morin

**Affiliations:** 1 Unité Virologie, Immunologie et Écotoxicologie des Poissons, Laboratoire de Ploufragan-Plouzané-Niort, National Infrastructure Emerg’In, Agence Nationale de Sécurité Sanitaire de l’Alimentation, de l’Environnement et du Travail (ANSES), 29280 Plouzané, France; lenaig.louboutin@anses.fr (L.L.); joellecabon@orange.fr (J.C.); 2 Unité Génétique virale et biosécurité, Laboratoire de Ploufragan-Plouzané-Niort, Agence Nationale de Sécurité Sanitaire de l’Alimentation, de l’Environnement et du Travail (ANSES), 22440 Ploufragan, France; veronique.beven@anses.fr (V.B.); edouard.hirchaud@anses.fr (E.H.)

**Keywords:** sea bass, mortality, genome description, sea bass toti-like virus (SBTLV), epidemiology

## Abstract

The European sea bass *Dicentrarchus labrax* is the main species reared in Mediterranean aquaculture. Its larval stage, which is very sensitive and highly affected by sanitary and environmental conditions, is particularly scrutinized in hatcheries. Recently, a Mediterranean sea bass farm had to deal with an abnormal increase in mortality, especially between 20 and 35 days post-hatching (dph). Biological investigations led to the observation of cytopathic effects on three different fish cell lines after almost 3 weeks of culture at 14 °C in contact with homogenized affected larvae, suggesting the presence of a viral agent. High-throughput sequencing revealed a 6818-nucleotide-long RNA genome with six putative ORFs, corresponding to the organization of viruses belonging to the *Totiviridae* family. This genome clustered with the newly described and suggested Pistolvirus genus, sharing 45.5% to 37.2% nucleotide identity with other piscine toti-like viruses such as *Cyclopterus lumpus* toti-like virus (CLuTLV) or piscine myocarditis virus (PMCV), respectively. Therefore, we propose to name this new viral agent sea bass toti-like virus (SBTLV). Specific real-time RT-PCR confirmed the presence of the viral genome in the affected larval homogenate from different production batches and the corresponding cell culture supernatant. Experimental infections performed on sea bass fingerlings did not induce mortality, although the virus could be detected in various organs and a specific immune response was developed. Additional studies are needed to understand the exact involvement of this virus in the mortality observed in hatcheries and the potential associated cofactors.

## 1. Introduction

The development of marine aquaculture over the last decades has been accompanied by several sanitary issues of importance that have caused economic losses owing to reduced growth performances, mortalities, or associated treatment costs at various stages of fish development [1].

The global burden of pathogens on European sea bass (*Dicentrarchus labrax*) production units in the Mediterranean Sea has recently been evaluated through the European project MedAid. Tenacibaculosis, vibriosis, and photobacteriosis were the most common bacteriosis reported, whereas the viral encephalopathy and retinopathy induced by the nervous necrosis virus (NNV) remained the main virosis [2].

If the on-growing stages are carried out in an open environment, reproduction and larval development are performed in hatcheries with high biosecurity levels, including management of water quality, regular sanitary controls, and vaccinations. Larvae are fragile, and their growth is associated with a significant level of mortality, especially during the first few days post-hatching (dph). Despite all the precautions taken, these mortality episodes can sometimes be more intense, with no identification of the exact cause(s) of this excess mortality. Viruses belonging to the *Totiviridae* family are known to infect a wide range of hosts, mainly unicellular-like protozoans or fungi [3,4]. Five genera have been recognized by the ICTV: *Giardiavirus*, *Leishmaniavirus*, *Trichomonasvirus*, *Totivirus*, and *Victorivirus*. The first three genera contain viruses infecting protozoa, whereas *Totivirus* and *Victorivirus* target yeast and fungi [3,5]. Numerous other viruses, qualified as “toti-like viruses” belonging to Totiviridae family, have been described in recent decades, but they have not been assigned to a specific genus. Among these, the infectious myonecrosis virus (IMNV), described in shrimp, clusters with some species of *Giardiavirus* but could represent a new genus within the family *Totiviridae* [6,7]. Several unassigned arthropod viruses have also been proposed to form a new genus called *Artivirus* [8], and totivirus-like viruses have also been described in ants, with *Camponotus yamaokai* virus being the first *Totiviridae* member reported in a hymenopteran [9]. Piscine myocarditis virus (PMCV), identified as the causative agent of cardiomyopathy syndrome (CMS) and responsible for high losses in salmon farms, notably in Scotland and Ireland, is another unclassified member of the *Totiviridae* family [10,11,12,13]. Recently, several new toti-like viruses have been identified in fish in the context of natural mortality events, notably owing to the generalization of high-throughput sequencing. This was the case for common carp toti-like virus-1 (CCTLV-1) in common carp, bluegill toti-like virus-1 (BGTLV-1) in bluegill, *Cyclopterus lupus* toti-like virus (CLuTLV) in lumpsucker fingerlings, or golden shiner toti-like virus-1 (GSTLV-1) in golden shiner *Notemigonus crysoleucas* baitfish [7,14]. Interestingly, none of the animal toti-like viruses reported in recent years could be assigned to one of the previously described genera of the family *Totiviridae*, suggesting the existence of other potentially unknown genera [4].

*Totiviridae* are non-enveloped double-stranded RNA viruses with an icosahedral capsid. Their genomes vary from 4600 to more than 8000 nucleotides long, coding at minimum for a capsid protein and an RNA-dependent RNA polymerase (RdRp) and, according to the genus, may contain variable number (2 to 4) of potential additional open reading frames (ORFs) [15]. Totiviridae translation of capsid and RdRp is characterized by the typical −1, −2, or +1 ribosomal frameshifting described in giardiaviruses, totiviruses, trichomonasviruses, and leishmaniaviruses, respectively; viruses belonging to these genera usually display the capsid–RdRp fusion protein. In contrast, capsid and RdRp are expressed separately after a termination/reinitiation process in Victoriviruses [16].

In the context of an increase in mortality/morbidity in several batches of a sea bass hatchery in winter 2018, we used a panel of cellular, serological, and molecular methods associated with in vivo experimental infections to characterize a virus isolated from symptomatic larvae. For this virus, which has never been described to date and has a 6818-nucleotide-long RNA genome structurally and phylogenetically assigned to the *Totiviridae* family, we propose the name sea bass toti-like virus (SBTLV).

## 2. Materials and Methods

### 2.1. Biological Samples

#### 2.1.1. Larvae and Juveniles

The first samples of clinically diseased sea bass larvae (3 batches, 22–27 dph), raised at a water temperature ranging from 18.5 °C to 20 °C, were collected in February 2018 (identified as QQ40, QQ41, and QQ42). Additional samples of sea bass or seabream larvae, sea bass eggs, rotifers, Artemia, or isolated internal sea bass organs with a healthy or moribund status were obtained at various times from 2018 to the end of 2019 (Table 1, samples used for initial diagnosis (*n* = 10); Table S1, samples used for epidemiological monitoring (*n* = 86)). Twenty-eight additional batches corresponding to 250 samples were also regularly analyzed from the beginning of 2021 to spring 2022. All samples were stored at a temperature lower than 10 °C until cell culture analysis (with a maximum of 72 h between sampling and analysis) or in an RNA stabilization reagent (RNAlater™, Invitrogen, Waltham, MA, USA) before being processed for RNA analyses.

For cell culture applications, approximately 1 g of larvae was homogenized in a mortar with pestles and sand and then transferred into 9 mL of Eagle’s solution containing antibiotics (200 IU·mL^−1^ penicillin G, 0.2 mg·mL^−1^ streptomycin, and 0.2 mg·mL^−1^ kanamycin). Samples were then centrifuged (2000× *g*) for 15 min at 5 °C +/− 3 °C and the supernatants were collected. For molecular applications, samples were homogenized in phosphate-buffered saline (PBS, pH 7.4, Merck, Darmstadt, Germany) using FastPrep-24™ 5 (MP Biomedicals™, Seven Hills, NSW, Australia) with a 10% weight/volume ratio.

#### 2.1.2. Genitors

Eggs and sperm or gonads from sea bass or seabream were sampled from various batches of mature fish. In order to improve the level of viral detection and the yield of extraction, samples were cleaned through successive steps of centrifugation and then concentrated. Briefly, 1 to 15 g of tissue was processed by adding 10 to 15 mL of PBS (0.2 M NaCl, 50 mM Tris-HCl, 5 mMCaCl2, 5 mM MgCl_2_, pH 7.5) per gram of tissue. Samples were frozen/thawed 3 times, centrifuged at 2000× *g*, and the supernatant mixed with 10 volumes of cold PBS. Samples were then serially centrifuged to remove tissue debris at 1000× *g*, 3000× *g*, 5000× *g*, 8000× *g*, 10,000× *g*, and 12,000× *g* for 5 min at 5 °C +/− 3 °C, discarding pellet and centrifuging the supernatant again for each step. The final supernatant was transferred into an ice bath for 10 min before laying it down on a sucrose cushion at 28% (*w*/*w*) and ultracentrifugation at 300,000× *g* for 2 h at 5 °C +/− 3 °C. The supernatant was discarded and the pellet mixed with 100 to 200 µL of cold PBS before use for nucleic acid extraction. Internal organs (kidney, spleen, heart) were processed in parallel (homogenization with mortar and pestle) without applying the purification/concentration protocol.

### 2.2. Cell Culture Isolation

For each sample analyzed, 100 µL of supernatant was inoculated onto 24-well-plate cultures of a total of 11 cell lines: bluegill fry (BF-2) [17], epithelioma papulosum cyprini (EPC) [18], chinook salmon embryo (CHSE-214) [19], rainbow trout gonad (RTG-2) [20], white sturgeon skin (WSSK-1) [21], koi carp fin (KF-1) [22], eel kidney-1 (EK-1) [23], striped snakehead (SSN-1) [24], sea bass larvae (SBL) [25] and larvae of baeri sturgeon (LEB, homemade). Inoculated cell cultures were incubated at 14 °C ± 2 °C and checked regularly for the development of a cytopathic effect (CPE). A blind passage was performed after 7 days on the same cell lines. When a CPE was observed, the cell supernatant was filtered and a chloroform test was performed to check for the presence of a lipid-bilayer-enveloped virus, as well as a specific detection for viral RNA using the double-stranded (ds) RNA antibody J2, as described by Louboutin et al. [26].

### 2.3. Production of Specific Rabbit Antibodies

Antibodies specific to the isolated virus were produced on specific pathogen-free rabbits immunized by four subcutaneous injections of 1 mL (days 0, 7, 14, and 36) of a 25% glycerol-cushion-purified virus from homogenized infected sea bass larvae (viral amount of around 6 × 10^11^ copies RNA·mL^−1^; rabbit n°183) or from clarified positive cell supernatant (viral amount of around 5 × 10^12^ copies RNA·mL^−1^; rabbit n°184; Biotem Company, Isère, France). Antibody titers of sera collected on D28, 42, and 56 were assessed using a homemade ELISA coated with shredded and purified infected larvae (Biotem protocol). Rabbit n°183 D42 sera was selected for the immunofluorescence antigen test (IFAT) and rabbit n°183 D56 sera was used for ELISA development.

### 2.4. Indirect Fluorescent Antibody Test (IFAT)

A specific IFAT was performed in cell cultures with CPEs using a specific polyclonal rabbit antibody n°183 D42 diluted 1:2000 in PBS-T20 (phosphate-buffered saline, 0.02% Tween 20).

### 2.5. Transmission Electronic Microscopy (TEM)

The isolated virus was propagated in a 25 cm^3^ flask (Clipmax, TPP, Geneva, Switzerland) on the LEB cell line at 14 °C. At day 2 pi, the medium was removed, infected as well as non-infected cells were washed with 0.1 M pH 7.2 cacodylate buffer, and then a fixation buffer (glutaraldehyde 2%–paraformaldehyde 2%–tampon cacodylate 0.1 M, pH 7.2) was added for 1 h at room temperature (RT). The cells were washed 3 times with cacodylate buffer and stored at 5 °C until processing. TEM was performed using the Merimage platform (Roscoff, France) and examined with a Jeol 1400 transmission electron microscope operating at 80 kV. Images were obtained using a Gatan (Pleasanton, CA, USA) Orius camera.

### 2.6. Nucleic Acid Extraction, Library Preparation, and High-Throughput Sequencing

RNA was extracted from either 100 to 200 µL larvae homogenates (RR200) or cell culture supernatants (QQ40 1st passage and QQ40 10th passage; see Table 1) using, respectively, a NucleoSpin virus kit (Macherey-Nagel, Hoerdt, France) or an Adiamag kit (Bio-X Diagnostics, Belgium) coupled with a KingFisher Duo Prime instrument (ThermoFisher Scientific Inc., Worcester, MA, USA) following the manufacturer’s instructions. cDNA libraries were prepared with an Ion Total RNA-Seq Kit (Life Technologies, Carlsbad, CA, USA) following supplier’s instructions. The cDNA libraries were sequenced using an Ion Proton Sequencer and Ion PI Chip v3 (Life Technologies, Carlsbad, CA, USA). The reads were first mapped onto the host’s genome using Bowtie 2 [27]. Unmapped reads were then assembled with SPades (v3.10.0, option-careful) [28] and the de novo contigs were BLASTed against the NCBI nr nucleotide or protein databases for identification of viral sequences.

### 2.7. Phylogenetic Analyses

The predicted ORFs of the contig coding for viral sequences were analyzed with Geneious (Geneious Prime^®^ 2020.2.4, Boston, MA, USA). To investigate the relationship between the isolated virus and other known viruses, protein sequences coding for RdRp were collected on Genbank and aligned with sequences of piscine totiviruses and other totiviruses or toti-like viruses using MAFFT version 7.221 https://doi.org/10.1093/molbev/mst010 (accessed on 24 February 2023); alignments were cleaned with Trimal (version 1.4.1; https://doi.org/10.1093/bioinformatics/btp348). Pairwise identity matrices were obtained using BioEdit (version 7.2.5.). Phylogenetic reconstructions were generated using maximum likelihood (ML) after selecting the best-fit model with IQ-TREE 2.1.2 COVID-edition built 30 March 2021 [29]. The LG + F + R9 substitution model was selected [30] and the ultrafast bootstrap approximation was used [31]. A phylogenic analysis with ORF3 SBTLV protein was also performed using the same strategy. The best-fit model according to AIC was WAG + F + G4. The phylogenetic trees were represented and annotated using iTOL [32].

### 2.8. PCR Tools for Viral Detection and Quantification

Several pairs of primers were designed along the viral genome to control the consistency of the sequences obtained by RNA high-throughput sequencing or after amplification of fragments and Sanger sequencing or to perform real-time RT-PCR (RT-qPCR, see Table 2 for details). RNA was extracted using the protocol described in Section 2.6. The fragments were amplified with a one-step conventional RT-PCR (RT-cPCR) reaction performed with the SuperScript III One-Step RT-PCR System with Platinum Taq High Fidelity (Invitrogen, Waltham, MA, USA) using the following mix: approximately 1 µg of RNA was added to 20 µM of each primer, 1 µL of Taq, 25 µL of reaction mix, and water in a final volume of 50 µL. The RT-cPCRs were conducted in a thermocycler T100 (Biorad, Hercules, CA, USA) with an initial step of 52 °C (30 min), followed by one step at 94 °C (2 min), then 40 cycles of 94 °C (15 s), 55 °C to 62.5 °C (30 s) depending on the hybridization temperature of the primers, and 68 °C for the elongation step (20 s to 60 s depending the fragment length; see Table 2). The sizes of the PCR products were assessed on agarose gel (2%). 5′ and 3′ rapid amplifications of cDNA end (RACE) PCRs using the 5′/3′ RACE Kit—2nd generation (Roche) were performed on a QQ40 sample to confirm the 5′ and 3′ sequences of the virus genomes following the manufacturer’s instructions. Briefly, a specific primer oPVP612 was used to produce complementary cDNA with a polyadenylated tail necessary for an oligo dT anchor (contained in the kit). Two successive PCRs using oPVP615 and then oPVP616 with the anchor primer were finally performed to obtain a fragment of 257 nt. 3′RACE was performed through the addition of a poly-A tail on viral RNA with DNA polymerase I (*E. coli*) (NEB, Hitchin, UK), cDNA synthesis, and a final PCR using the primer oPVP606 (position 6377, Table 2). All PCR products generated were purified using a NucleoSpin gel and PCR clean-up kit (Macherey-Nagel) and cloned using the TOPO TA cloning kit (Invitrogen). Three clones were selected and sequenced in both directions, and all nucleotide differences were checked visually using VectorNTI v.11.5 software.

An additional set of primers, oPVP559 5′-CCGAGGCTATCAAAGTCAGC-3′ and oPVP560 5′-GCAGTCCATCTCCAACCACT-3′, located at position 3920 to 4029 (fragment length 110 bp) of the virus genome was designed (Primer3plus) for a specific RT-qPCR. Reverse transcription (RT) and amplification were performed using the SuperScript III One-Step RT-qPCR System (Invitrogen, France) using the following mix: 5 µL of extracted RNA was added to 800 nM of each primer, 300 nM of probe, 0.5 μL of Invitrogen RT mix, 12.5 μL of reaction mix (2×), and water in a final volume of 25 μL. To assess the effectiveness of the whole analytical process, a known amount of external exogenous control (RNA bacteriophage MS2) was spiked into the samples before lysis. Nucleic acid extraction and specific RT-qPCR [33] were performed in parallel with the targeted virus. The RT-PCRs were conducted in a QuantStudio5 (Applied, Cleveland, OH, USA) after an initial step of denaturation at 95 °C (5 min), with an initial step at 50 °C (30 min), followed by one step at 95 °C (15 min), then 40 cycles at 94 °C (15 s) and 60 °C (60 s). Results were expressed as cycle threshold (Ct), and the Ct values obtained for MS2 were systematically reported in predefined control cards with low and high values to validate the analyses. A PCR product of 286 nucleotides long, covering the RT-qPCR zone, and containing the specific T7 sequence, was obtained with the primers oPVP 608 and 609. This product was used as a template for in vitro synthesis of an RNA transcript using T7 RNA polymerase (T7 RiboMAX™ Express Large Scale RNA Production System, Promega, Madison, WI, USA) following the manufacturer’s recommendations. Quantification via spectrophotometry (Nanodrop One, Thermofisher, Waltham, MA, USA) gave a titer of 2 × 10^13^ RNA copies.µL^−1^. Aliquots were stored at −80 °C and diluted in 10-fold dilutions for standard curve use and absolute quantification of the viral amount in samples (expressed as number of copies·mg tissue^−1^) and also included in each run as a positive amplification control.

### 2.9. Data Availability

The virus genome sequences are available in GenBank under the following nucleotide accession numbers: OQ791576 for QQ40-1st passage on BF_2_, OQ791577 for RR200-MINOR, OQ791578 for RR200-MAJOR, and OQ791581 for QQ40-10th passage on LEB cells. Sequencing data were submitted to the NCBI Sequence Read Archive and are available under the BioProject accession numbers listed in Table 3.

### 2.10. Experimental Trials

#### 2.10.1. Fish

A total of 125 sea bass fingerlings (3.0 to 8.5 g) and 20 adults (mean weight 400 g) were used through 3 different protocols. Before the experiments, some individuals from each batch underwent virological (inoculation on EPC, BF2, and SSN-1 cell lines) and bacteriological (nonspecific medium) analyses to confirm the absence of cultivable and pathogenic agents.

#### 2.10.2. Experimental Design

##### Authorization to Conduct Research and Ethical Aspects

All the protocols were conducted in accordance with European Commission Recommendation 2007/526/EC on revised guidelines for the accommodation and care of animals used for experimental and other scientific purposes. The ANSES Plouzané site has authorization to conduct experiments on fish in its facilities according to Administrative Order No. C29-212-3 issued by the Prefecture of the Finistère. Furthermore, the procedure was approved by the national ethics committee on animal experimentation (COMETH ANSES/ENVA/UPC No. 16) and was authorized by the French Ministry for Education, Higher Education and Research under No. 20-023 #24166. Euthanasia involved the addition of a lethal dose of 100 ppm of Eugenol into the tank water. Animals were kept in contact with Eugenol until the complete disappearance of any respiratory activity. In case of evident signs of suffering during the experiments, compassionate euthanasia was performed.

##### Experimental System

Assays were carried out in filtered and UV-treated seawater continuously flowing into each tank (open circuit) to maintain optimal water conditions for the fish throughout the experiments (oxygen saturation > 80%, pH close to 8, water free of nitrates and nitrites). The tanks were maintained in a natural light/dark cycle (14 h/10 h in winter) in a room with air volume renewed every hour. The water temperature was either unregulated (first assay) or regulated at 18 °C ± 2 °C (second assay) or 20 °C ± 2 °C (third assay) and recorded with a wireless probe (Cobalt, Oceasoft^®^, Dickson, Addison, USA) coupled with an acquisition system (ThermoClient 4.1.0.24). For the whole experiments, fish were fed twice a day with commercial mini-pellets NeoGrower extra Marin (Le Gouessant Aquaculture, Lamballe, France).

##### Viral Challenge Conditions

In a first assay, 70 fingerlings were infected in a 50 L (L) tank. A total of 50 were immersed for three hours in 3 L of hyperoxygenated seawater containing 10 mL of the isolated virus (9th passage on LEB, with notable CPEs), whereas 20 were injected intraperitoneally (IP) with 50 µL of the same viral suspension and positioned in a basket in the same tank. Fifty negative controls were positioned in another 50 L tank after being immersed in Eagle’s solution containing antibiotics instead of the virus suspension. General behavior, the presence of clinical signs, and mortalities were recorded daily. At 16, 23, and 30 days post-infection (dpi), 5 fish per condition (negative controls were only sampled at 16 dpi) were sacrificed and the following organs were sampled: blood, brain, kidney, heart, spleen, dorsal fin, and gills. At the end of the experiment (60 dpi), three fish (immersed or injected) were sampled. Survivors were euthanized following the procedure described before.

For the second assay, 55 fingerlings, reared in a 50 L tank, were IP infected with viral supernatant (100 µL of an 11th passage on an LEB cell line with CPEs). The pelvic fins, dorsal fins, gills, hearts, spleens, and kidneys from five fish were sampled at 1, 4, 7, 12, 19, 26, and 35 dpi. In parallel, blood from the same fish was individually sampled for ELISA assay. Final blood sampling was performed at 4 months p.i.

The last assay included 20 adults. Seven fish were IP injected with 1 mL of the virus supernatant (23rd passage on the LEB cell line, controlled via RT-qPCR, Ct = 16.06) and seven were injected with a larvae homogenate (controlled by RT-qPCR, Ct = 20.44). Six fish were used as negative controls. Fish were positioned in three 400 L tanks and fed and monitored for more than 2 months. At 17 dpi, fish were anesthetized with Eugenol (20 ppm) and a small amount of blood was withdrawn from the caudal vein with a lithium heparinized vacutainer (BD Vacutainer LH 85 IU). At 73 dpi, a final sampling was performed with a maximum of blood withdrawn. Blood was centrifuged at 1200× *g* for 10 min, and the plasma samples were stored at −80 °C for further ELISA applications.

### 2.11. ELISA Test

Plasma samples were obtained either after several experimental trials conducted at Anses (see Section Viral Challenge Conditions) or directly from the infected farm.

Maxisorb^®^ 96-well polystyrene plates (Nunc Immunoplates, Rochester, USA) coated with 100 µL·well^−1^ of non-purified rabbit anti-virus IgG n°183 D56 diluted 1/1000 in PBS were incubated for 16 h at 5 °C ± 3 °C. Twin plates, i.e., one with the virus and one as a control in which cell culture supernatant was used instead of the viral suspension, were prepared. Plates were washed 3 times with washing buffer (PBS-T20; phosphate-buffered saline, 0.02% Tween 20). Non-specific sites were blocked with homemade blocking buffer BB (5% skimmed milk, 0.2% Tween 20, 0.01% thimerosal mixed in PBS) and the plates incubated for 1 h at 37 °C before washing twice with PBS-T20. Then, 100 μL of viral suspension diluted to 1/2 in diluting buffer (DB; BB diluted to 1/10 in PBS) was distributed into each well and the plates incubated for 1 h at 37 °C before being washed three times with PBS-T20. A volume of 100 μL of sea bass plasma diluted to 1/100 in diluting buffer was distributed into each well, and the plates were then incubated for 2 h at 20 °C. Positive and negative control plasma obtained from in vivo assays were added to each plate. After washing three times with PBS-T20, the plates were incubated with 100 μL of purified and biotinylated rabbit anti-sea bass immunoglobulin M (IgG anti-IgM) diluted to 1/2000 in DB. After 1 h incubation at 37 °C, the plates were washed four times with PBS-T20. The wells were then incubated for 1 h at 37 °C with 100 μL of ExtrAvidin^®^-Peroxydase (Sigma, Burbank, CA, USA) diluted to 1/1000 in DB and washed six times with PBS-T20. A volume of 100 μL of a solution containing 4 mg of O-phenylenediamine (Sigma) and 4 μL of 30% hydrogen peroxide (Sigma) in 10 mL phosphate citrate buffer (pH 5) were distributed in each well for the revelation of the binding, and the plates were incubated for 20 min at RT. Finally, 25 μL of H_2_SO_4_ 3 M (ChemLab) was added to each well to stop the reaction, and absorbance was measured spectrophotometrically at 492 nm in a Spark 10 M Tecan analyzer (Tecan Trading AG, Männedorf, Switzerland). The absorbance differences (ΔOD) between the wells with and without the virus were calculated, and the relative quantity of antibodies was expressed as the percentage of the relative value compared with the ΔOD of the positive control. The plasma was considered positive when the difference was greater than the threshold of positivity (TP) calculated as: TP = mean percentage of the negative control of the test + three standard deviations (SD) defined from 38 plasma samples from virus-free sea bass.

### 2.12. Statistical Analysis

All statistical analyses were performed using XLSTAT statistical software (2020), with a significance level of 5%. Viral load values were compared between healthy and dying larvae in the first instance and regarding the age of larvae in the second instance using a Mann–Whitney non-parametric test. All data are expressed as the mean ± standard deviation (SD).

## 3. Results

### 3.1. Clinical Signs in Hatchery and First Laboratory Investigations

Clinical signs of exacerbated nervous behavior and/or an increase in mortality was observed 22–27 dph in the first samples of sea bass larvae analyzed in 2018 (QQ40, QQ41, and QQ42; Table 1); these were signs that actually triggered the analyses. Similar behaviors for the larvae were observed in 2019 on 22–30 dph larvae from other batches, but with less consistent symptoms and reduced mortalities. Mortalities between 26 and 32 dph could reach up to 30% of the batch, whereas in some batches there were no clinical signs and high survival at the weaning stage. There were no reports of clinical signs or mortalities before 22 dph or beyond 35 dph.

Apart from sporadic detection of environmental *Vibrio* sp., bacteriological analysis did not reveal the presence of any known pathogens in tissues sampled from either dead or moribund fish during these two years. In the same way, no specific histological changes were detected in larval brains; there was only mild vacuolization in hepatocytes in a limited number of cases.

### 3.2. Virus Isolation

**Figure 1:**
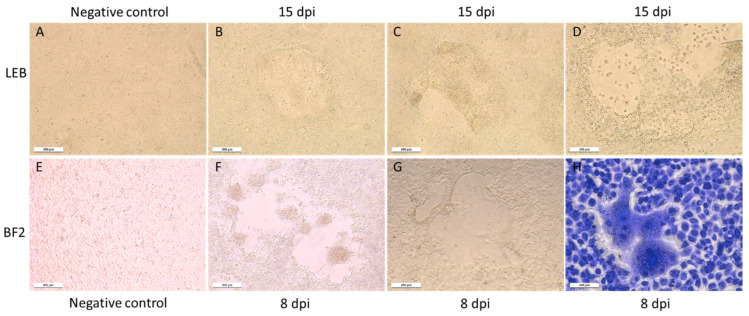
CPEs in infected cell cultures. (**A**–**D**) LEB cell line. (**A**) Non-infected cells at 15 dpi; (**B**–**D**) various sizes of CPE observed 15 dpi with positive toti-like virus supernatant. (**E**–**H**) BF2 cell line. (**E**) Non-infected cells 15 dpi; (**F**,**G**) infected cells at 8 dpi with extension of a CPE characterized by an enlargement of the hole in the monolayer. The CPE led to the production of “bubbles” that come off the monolayer. (**H**) Observation of syncytia after Giemsa coloration at 8 dpi.

**Figure 2:**
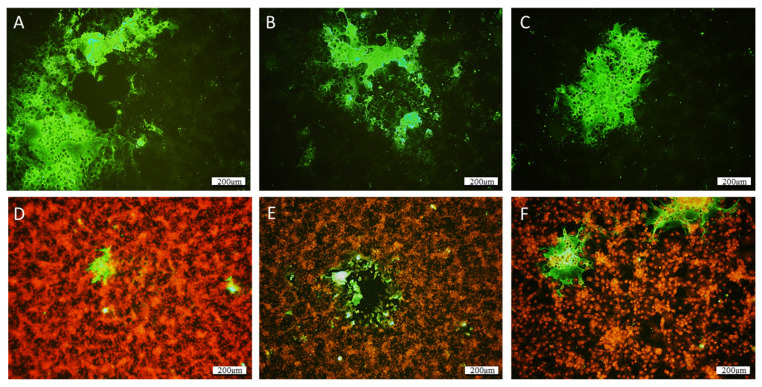
IFAT reaction on cell culture presenting CPE using a commercial J2 monoclonal antibody targeting dsRNA. Infected cells are characterized by green-fluorescent cytoplasm. Large syncytia are easily distinguishable by the fusion of numerous cells, which form plurinuclear large cells. (**A**–**C**) Without counterstaining. (**D**–**F**) Nuclei were counterstained with propidium iodide. In (**D**–**F**), infected cells can be observed, notably next to holes in the monolayer. Scale bar: 200 µm.

Atypical CPEs, with enlarged cells with granular cytoplasm, were observed 3 weeks after the inoculation of the BF2, LEB, and WSSK-1 cell lines at 14 °C with homogenates of the affected larvae identified as QQ40, QQ41, and QQ42. The CPE evolved to form very large syncytia and led to a slight destruction of the cell monolayer (Figure 1). None of the other cell lines tested displayed CPEs. The IFAT, performed using J2 antibody, revealed the presence of dsRNA in infected cells, whereas no fluorescence was detected in the negative controls (Figure 2).

Susceptible cell lines inoculated with CPE+ culture supernatant displayed accelerated an CPE over the in vitro passages, with appearance of CPEs as early as 7 dpi in LEB cells and 2–3 dpi on BF2 cells after 3 and 2 passages, respectively. Treatment of inoculated cell culture supernatants with chloroform before a new blind passage had no effect on the appearance of CPEs.

In March, April, and November 2019, additional samples from either healthy (QQ112, QQ118, QQ119) or symptomatic (QQ111, RR200, SS200, SS52; Table 1) larvae were processed via cell culture and led in all the cases to the observation of similar CPEs (Figure 1).

### 3.3. Electron Microscopy

**Figure 3:**
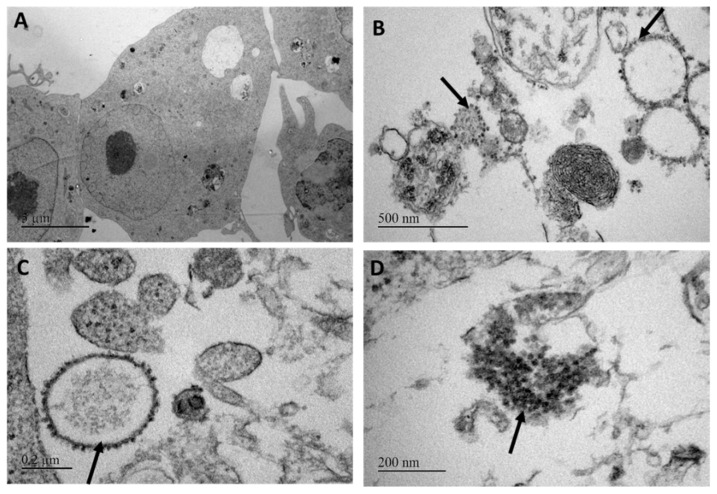
TEM of LEB cells infected with a cell culture supernatant positive for toti-like virus (isolation from sample QQ40). (**A**) Negative control; (**B**–**D**) infected LEB cells at 2 dpi. Spherical particles budding from vacuoles are shown with black arrows.

Some electron-dense particles, which were around 30–40 nm in diameter, were observed in LEB cells harboring a large CPE after 7–8 days of incubation (Figure 3) and had a location compatible with an excretion from some organelles. These particles were not observed in uninfected LEB cells.

### 3.4. High-Throughput Sequencing of the Viral Genome and ORF Organization

RNA extracted from BF2 (sample QQ40 1st passage) and LEB positive cell supernatants (sample QQ40 10th passage), as well as from larvae homogenates from clinically infected batches (RR200), were processed for high-throughput sequencing with an Ion Torrent sequencing machine.

From samples RR200 and QQ40_BF2, after SPades assembly, two contigs of 6610 and 6834 nt long, respectively, with no known homology after a BLAST search on the NCBI-nt database, were identified. A Blastx on the NCBI nr protein database revealed a 42.87% identity with the *Cyclopterus lumpus* toti-like virus.

Subsequent Sanger sequencing and 5′ and 3′RACE PCRs confirmed some ambiguous nucleotides and precisely defined the extremities of the genome of the QQ40-BF2 sample to a final genome size of 6818 nucleotides. Further analyses of the RR200 samples by realigning the reads on the 6610 nt contig revealed significant heterogeneities in the consensus sequence that led to the identification of two variants, named RR200-MAJOR and RR200-MINOR, that presented 97.8% nucleotide identity with each other (a difference of 147 SNPs on 6610 nt). The MAJOR and the MINOR variants displayed 99.8% and 97.9% nucleotide identities with QQ40-BF2, respectively. The shorter sequences observed for the RR200 variants are explained by a lower deepness in reads and a loss of coverage of the 5′ and 3′ ends (no RACE was performed on these samples).

**Figure 4:**
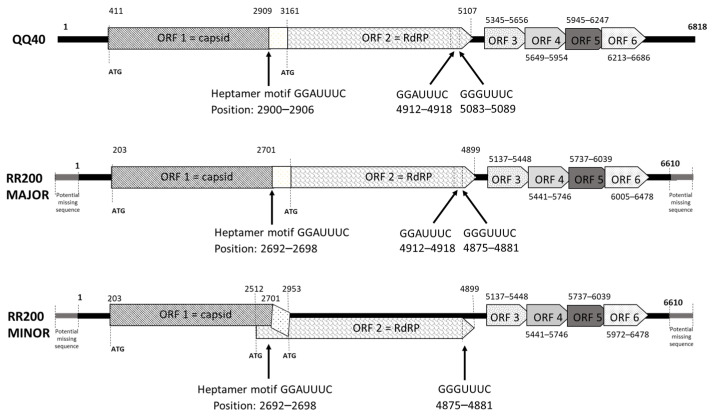
New toti-like virus genome organization. Putative open reading frames (ORFs, beginning and final nucleotides) and heptamer motifs are positioned on the sequence of the QQ40 sample and the RR200-MAJOR and MINOR variants; ORFs are suggested by ORFfinder. Putative ATG start codons are included with positions. RR200-MAJOR and MINOR are expected to be incomplete at the 5′ and 3′ ends.

The resulting consensus sequences corresponding to either the QQ40 supernatant or the RR200-MAJOR or RR200-MINOR homogenates were subjected to ORF prediction using ORFfinder. Regarding QQ40, six ORFs were proposed (Figure 4): ORF1, from nt position 411 to 2909 (frame 3); ORF2, from 3161 to 5107 (frame 2); ORF 3, from 5345 to 5656 (frame 2); ORF4, from 5649 to 5954 (frame 3); ORF5, from 5945 to 6247 (frame 2); and ORF6, from 6213 to 6686 (frame 3). The four putative distal ORFs slightly overlap. Blastp, performed on ORF1, revealed an amino acid (aa) identity of 42.3% with the capsid protein of the *Cyclopterus lumpus* toti-like virus (QZA60158.1, 353 identical aa/835) and with the putative polyprotein of *Cyclopterus lumpus* toti-like virus (QZA60159.1, 381 identical aa/648). ORF2 has strong homology with the RNA-dependent RNA polymerase (RdRp) of various toti-like viruses, the strongest identity (58.8%) again being with the putative polyprotein of *Cyclopterus lumpus* toti-like virus (QZA60159.1, 381 identical aa/648). As described for several toti-like viruses, a slippery site (GGAUUUC) is present six nucleotides before the stop codon of the ORF1 sequence. This frameshift site results in the re-engagement of the translational tRNAs in the −1 frame, allowing the production of ORF1 and ORF2 as a fusion protein. The protein ORF3 displayed 45% identity with the ORF3 of *Cyclopterus lumpus* toti-like virus (37 aa/82 with QYI86730.1) and also, it should be noted, a lower identity (35.71%) with a protein (p10) described from bat coronavirus (i.e., 20 aa/56 with AVP25418.1). ORFs 4, 5, and 6 presented no similarity with published protein sequences in the NCBI protein database. For sequences corresponding to the RR200-MAJOR and MINOR homogenates, the predicted ORFs were almost the same as those for QQ40, with the exception of ORF2 in the variant RR200-MINOR, which presented an ATG codon in frame with the expected ATG (compared with QQ40 sequences) but localized 441 nucleotides upstream. This would overlap with ORF1 by 190 nucleotides if this ATG was the initiation codon of the translation; the RR200-MINOR ORF2 would then be 795aa long (Figure 4). However, the slippery site at the 3′ end of ORF1 is still present and the translation of ORF2 as a fusion protein with ORF1 remains most likely. Two additional potential slippery sequences were also identified in QQ40-BF2 and RR200-MAJOR (Figure 4, only one in RR200-MINOR).

### 3.5. Phylogenetic Analysis

**Figure 5:**
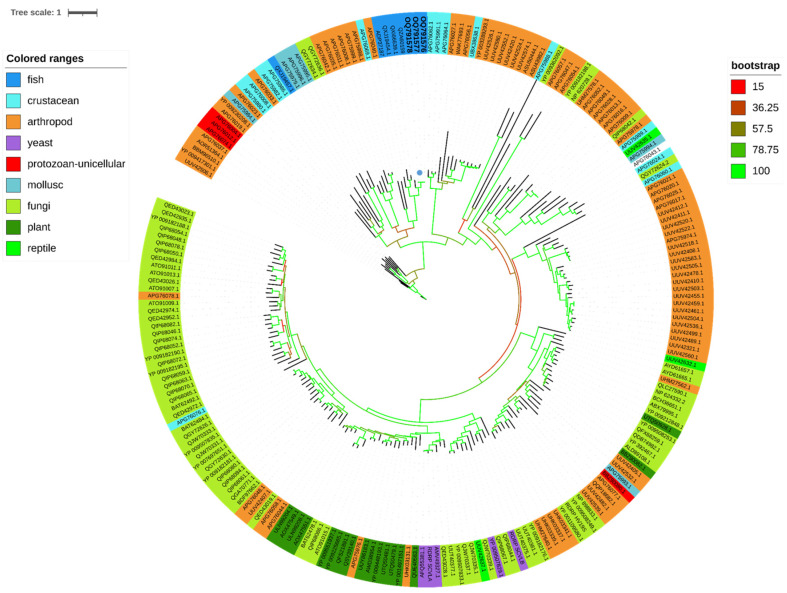
Maximum likelihood (ML) phylogenetic tree representing a large panel of viruses belonging to the *Totiviridae* family. Using a phylogenetic tree based on 305 amino acids of ORF2 (RdRp gene) with 219 sequences, the LG + F + R9 substitution model was selected and the ultrafast bootstrap approximation (1000 replicates) was used. SBTLV samples (QQ40/OQ791576, RR200-MAJOR/OQ791578, RR200-MINOR/OQ791577) are in bold and their positions are labelled using a solid blue circle. The color strip highlights the various host ranges on which toti and toti-like viruses were detected. The values for bootstrap support are represented by color codification, i.e., red for values from 0 to 15 and green for values from 78.75 to 100.

A phylogenetic tree was reconstructed based on ORF2, i.e., the RdRp sequence (Figure 5). Regarding ORF2, the newly described toti-like virus clustered with the lumpsucker toti-like virus (accession number QZA60159), the piscine myocarditis virus (PMCV; ADP37187), the common carp toti-like virus 1 (QXJ19454), and the golden shiner toti-like virus 1 (QXI66639). This group of piscine totiviruses differs from the bluegill toti-like virus 1 (QXJ19457), which clusters with several crustacean toti-like viruses (APG75982) and other arthropod-borne totiviruses, as well as mollusc toti-like viruses (accession numbers APG75996, APG75979, and APG75995).

**Figure 6:**
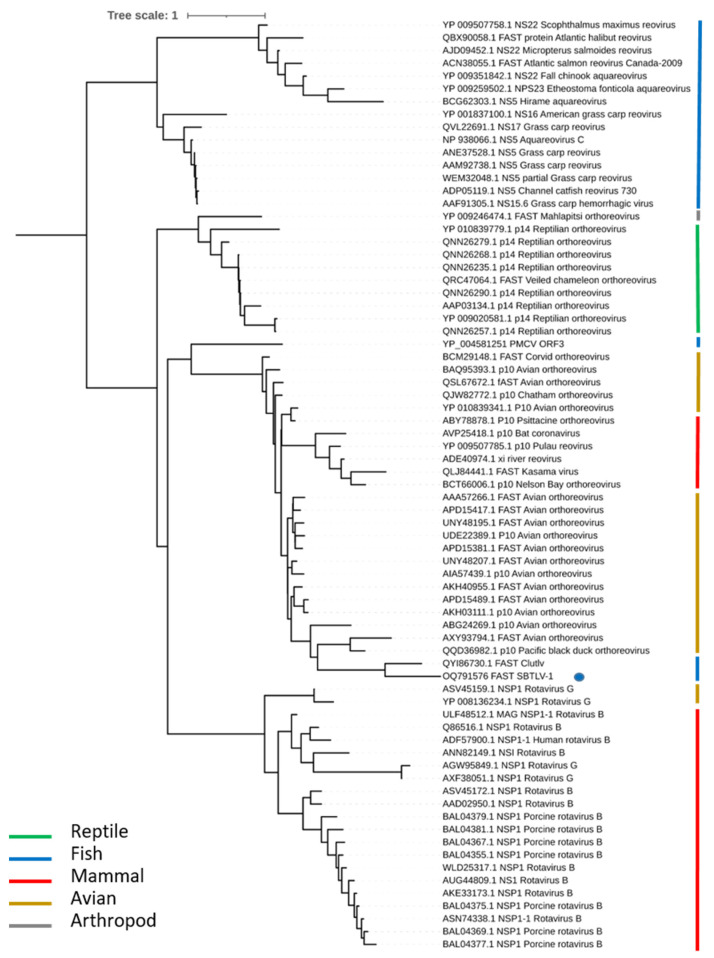
Phylogenetic position of FAST proteins. Phylogeny was estimated using a maximum likelihood method and mid-point rooted for clarity only. The virus described in this study is labelled using a solid blue circle. All horizontal branch lengths are scaled to the number of amino acid substitutions per site.

A phylogenetic analysis was also performed with the ORF3 proteins of SBTLV and CLuTLV (Figure 6). These two ORF3s share 46% identities and display similarities with FAST proteins described in two families of the Reovirales order, the Sedoreoviridae with some rotavirus B and G, and the Spinareoviridae with the Ortho- and Aquareovirus. The neighbor joining tree constructed with the FAST protein sequences from representatives of the different viral genera displayed four distinct clades, one for the rotavirus from birds and mammals, one for the Aquareovirus, one for the reptile Orthoreovirus, and the last one for the avian Orthoreovirus. Both FAST proteins from SBTLV and CluTLV cluster in the avian Orthoreovirus clade, as do the FAST sequences from the bat reovirus (and one coronavirus). It should be noted that the PMCV-FAST protein also clusters with the avian clade but, contrarily to SBTLV- and CluTLV-FAST proteins, in a very basal position.

### 3.6. Experimental Infections

#### 3.6.1. Virus Tropism and Spread

Neither clinical signs nor mortality were observed in our first assay, where fingerlings were infected by immersion or IP injection and followed up to 30 days, with sampling for analyses at 16, 23, and 30 dpi. No virus could be detected in organs collected from negative control fish at 16 dpi (Table 4). For infection by immersion, spleen and gills exhibited a positive RT-qPCR signal specific for totivirus at 16 dpi, the dorsal fin at 23 dpi (Ct = 35.99) only, and no signal was detected by RT-qPCR later on. For infection by IP injection, the brain was negative at 16 dpi and blood and brain at 60 dpi (Table 4); all the other organs tested were positive at all sample points. The earliest Cts were noticed for internal organs such as the kidney, spleen, and heart (ranging from 28.35 to 32.46). Taking into account the four sampling times, the viral RNA amount in the heart appeared to be significantly different from that in the kidney and spleen (*p*-value 0.004) and from the fins and gills (*p*-value 0.017), and the viral load in the spleen was significantly different from that in the kidney (*p*-value 0.001), heart (0.004), and fins and gills (0.017). Nevertheless, the dorsal fin exhibited earlier detection at 30 dpi (Ct = 29.67) compared with 16 dpi (Ct = 34.51).

**Figure 7:**
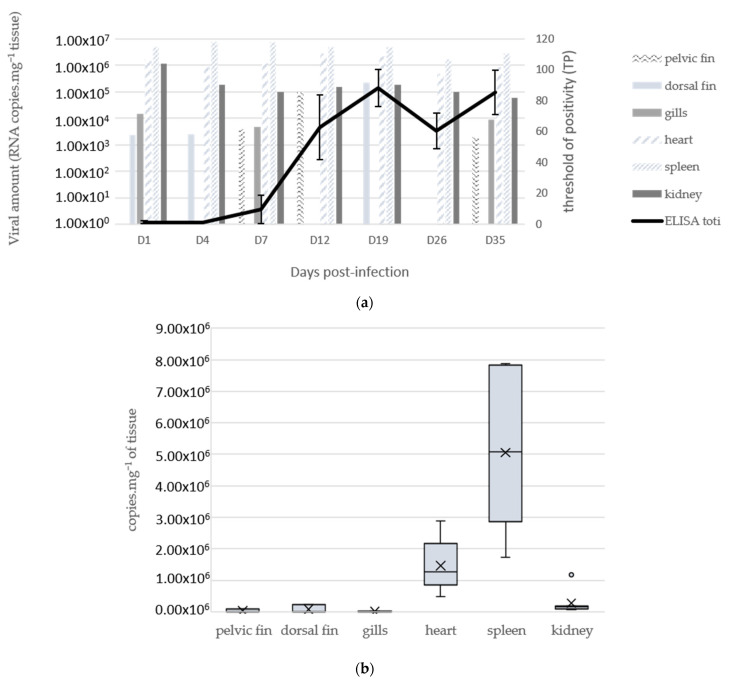
Viral loads per organ and specific antibody response after IP inoculation of fingerling sea bass with the toti-like virus. Fish were IP injected with toti-like-virus-positive supernatant and periodically sampled from 1 to 35 dpi. (**a**) Kinetics of viral loads per organ and antibody levels in blood. Viral loads were assessed by specific RT-qPCR on each organ pooled from five fish and are expressed as number of viral copies·mg^−1^ of larvae tissue. Specific antibody titers were determined through ELISA displayed on individual sera. (**b**) Global quantity of RNA copies·mg^−1^ of tissue in the different organs by cumulating all sampling times. X represent medians and horizontal bars means.

In the second assay, the IP route of infection was preferred, with more frequent sampling (1, 4, 7, 12, 19, 26, and 35 dpi). The toti-like virus was detected regularly in the different organs and at the different times tested (Figure 7a,b). Higher viral RNA amounts were obtained in internal organs (spleen, heart, kidney), and the virus was not systematically detected in fins. Significant differences were observed between the spleen (mean viral amount 5.0 × 10^6^ copies·mg^−1^ of tissue; *p*-value range from 0.001 to 0.017) or heart (1.5 × 10^6^ copies·mg^−1^ of tissue; *p*-value range from 0.004 to 0.017) and the other organs.

#### 3.6.2. Specific Antibody Response

Individual blood sampling was performed at each sampling time in the second experimental assay and plasma (*n* = 5 per condition) was analyzed using ELISA. The plasma samples from 1 and 4 dpi were negative. At 7 dpi, only one sample was positive. From 12 to 35 dpi, all plasma samples were positive, with an increasing signal from 12 to 19 dpi and then a stabilization (Figure 7a). After 4 months, 7 out of 8 fish still had a seropositive status.

For the third assay, all plasma samples collected from infected fish at 17 dpi and at 73 dpi revealed positive signals through ELISA assay (7/7 for IP with QQ40 and with SS200), whereas the three control fish tested were negative.

### 3.7. Screening of the Infected Farm

#### 3.7.1. Larvae Screening

**Figure 8:**
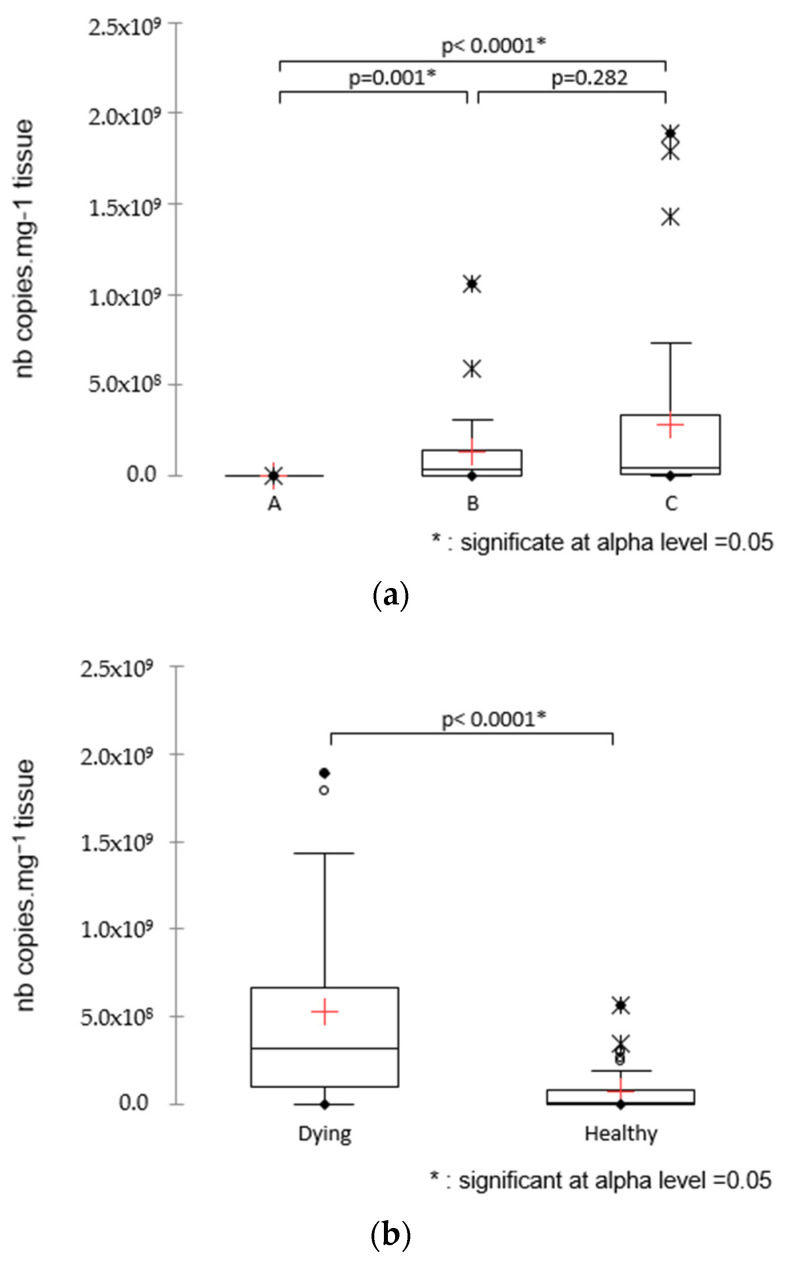

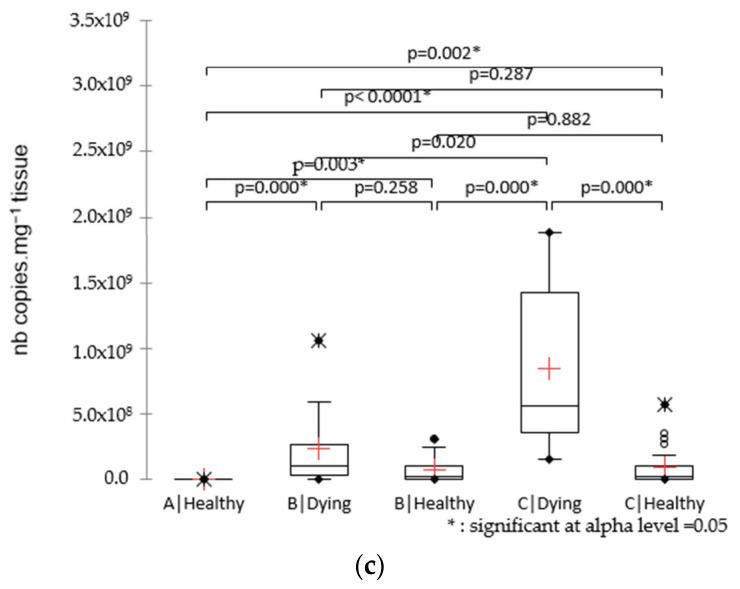
Viral load monitoring in larvae from 5 to 53 dph in various batches from the infected farm. A total of 70 samples were analyzed, with 6 samples from group A (status healthy), 29 from group B (10 dying, 19 healthy), and 35 from group C (9 dying, 26 healthy). Comparison of viral amount regarding the age of larvae (**a**); the health status (**b**); and the combination of the two parameters (**c**). Stars indicate a statistical difference between two conditions (Kruskal–Wallis; *p* < 0.05). + represent medians and horizontal bars means.

In 2019, 80 samples of sea bass (larvae aged of 5 days to 53 days (*n* = 72), eggs (*n* = 2), isolated internal organs from 79 dph larvae (*n* = 6)), as well as samples from seabream larvae (*n* = 4)), were analyzed through specific RT-qPCR. No SBTLV could be detected on sea bass larvae before 20 dph. The viral load seemed to increase from 20 dph to 25–30 dph, and various viral loads were observed depending on the batches of larvae and considering their age as well as their health status (Table S2 in Supplementary Materials). Three groups were defined: A for larvae younger than 20 dph, B for intermediate stages (20 < dph < 30), and C for the older ones (>30 dph). Significantly different viral loads were observed when comparing younger larvae (group A) with both older groups B and C (*p*-value < 0.0001), whereas intermediate larvae B and older ones C did not display a significant difference (*p*-value 0.077; Figure 8a). Regarding the health status, a significant difference was observed between healthy and moribund larvae, regardless of their age (*p*-value < 0.0001; Figure 8b); this significant difference also existed within group C (*p*-value 0.001; Figure 8c).

More than 250 samples of larvae, from 5 dph to 55 dph, were then regularly sampled in 2021 and 2022 and analyzed through RT-qPCR targeting toti-like virus. No correlation was found between the viral loads on days 25–30 and the survival rates of the batches. In parallel, we sporadically obtained high Ct values in seabream samples despite the absence of clinical signs.

#### 3.7.2. Broodstock Screening

Eggs and milt obtained from different batches of mature sea bass or seabream were analyzed using RT-qPCR. For the first 10 sea bass samples assessed, SBTLV was detected in seven batches after several steps of purification/concentration, as well as in 2 out of 5 seabream samples, whereas no signal was obtained using untreated extracts (see Table 5). Fourteen additional samples were processed and SBTLV detection was compared between organs and gonads when available. Twelve out of 14 samples displayed positive signals (Ct ranging from 31.5 to 39.3, thus 1.4 × 10^5^ to 2.1 × 10^2^ copies.reaction^−1^) in the gonads but appeared negative for internal organs.

## 4. Discussion

The purpose of this project was to identify the etiology of an abnormal mortality event observed in 20–35-day-post-hatching sea bass larvae from a hatchery. Cell culture inoculation with larval homogenates produced a CPE in cell lines, allowing the successful isolation of a virus, its re-inoculation into sea bass, and the investigation of its potential pathogeny in the larval and adult stages. The full-length genome of the virus was obtained using NGS and identified as a new virus related to the family *Totiviridae*. We propose the name sea bass toti-like virus (SBTLV) for the virus, a potential new member of the *Totiviridae* family.

Viral amplification in the LEB and BF2 cell lines was a several week process, and infected plates had to be trypsinized and replated with uninfected cells several times to increase the CPE and viral multiplication rate. Finally, the virus was isolated from healthy larvae after a long cultivation period in the BF2 and LEB cell lines. The presence of dsRNA, a hallmark of RNA virus replication, was confirmed via IFAT using the J2 antibody. Furthermore, a CPE-positive cell culture with a chloroform-treated inoculum was a feature of the presence of non-enveloped virions. The difficulty of viral amplification has also been reported for PMCV, another member of the *Totiviridae* family, which uses an extracellular transmission route for replication to spread to uninfected cells during cell division, sporogenesis, or cell fusion [11,34].

Our analysis, including NGS performed on the positive cell culture supernatant, revealed that the virus obtained from the QQ40 sample was an RNA virus with a genome size of 6.8 kb. Bioinformatics analysis of the sequence enabled the determination of six potential ORFs on the positive strand of viral RNA. The genomic organization is compatible with those of most of the *Totiviridae* family members, with a programmed ribosome frameshift (PRF) between the capsid (ORF1) and the RdRp (ORF2) leading to the production of a fusion protein that is thought to contribute to the correct encapsidation of the newly produced positive-strand RNA for the virion of most of the *Totiviridae* family members [35]. The phylogenetic analysis of the SBTLV RdRp attributes this protein to the Pistolvirus cluster proposed by Sandlund et al. [14], which represents viruses from fish species; however, it should be noted that not all fish toti-like viruses are members of this cluster, the golden shiner toti-like virus and the bluegill toti-like virus RdRps cluster with the toti-like viruses from arthropods.

Although Totiviridae have been initially described with only two proteins, the capsid and RdRp, additional proteins have been described in Toti–like viruses from various species, particularly from fish, with the number of additional proteins varying from one in PMCV, GSTLV-1, and CCTLV-1 to three in CluTLV [14] and four in SBTLV. The expression of these additional proteins and the mechanisms by which they are expressed deserves to be analyzed further. PMCV and SBTLV both display potential ribosome slippery sites upstream of the stop codon of ORF2; however, the downstream sequences have several additional stop codons in either of the three different frames, rendering this mechanism of translation unlikely.

In contrast with the ORF3s from PMCV, GSTLV-1, and CCTLV-1, which share almost no identity with other known proteins but might share structural properties and possibly have a chemokine-like domain [14], the SBTLV-ORF3 protein has a significant identity (45%) with the CLuTLV-ORF3 protein but also, to a lesser extent, with small p10 proteins found in some bat coronaviruses. CLuTLV-ORF3 displays a 40 to 50% identity with p10 proteins found in avian reoviruses. All of these p10 proteins, either from reovirus or coronavirus, are related to the fusion-associated small transmembrane (FAST) protein family, which mediates cell–cell fusion and syncytia formation [36,37,38]. The formation of syncytia observed in SBTLV-infected cells is probably mediated by the proteins coded by ORF3. The phylogenetic analysis of the FAST proteins from Reovirales highlights four distinct clades corresponding to fish, reptiles, and avian Orthoreovirus and the fourth clade corresponding to rotaviruses. The FAST proteins found in fish totiviruses belong to the avian clade, suggesting a recombination event in the FAST sequence from an avian reovirus and not from a fish reovirus, as might be expected. The PMCV FAST protein does not cluster with SBTLV or CLuTLV, suggesting distinct events leading to acquisitions of these FAST proteins in fish totiviruses.

Experimental infections were performed to assess the tropism and pathogeny of the newly isolated SBTLV and to characterize the development of an immune response towards SBTLV. Even if SBTLV could be detected in sampled fish using RT-qPCR after infection via immersion or intraperitoneal injection, no clinical signs nor mortality were observed in the fingerlings. The SBTLV-positive fish from the immersion condition indicated that the virus could enter fish through a natural route of infection and persist in infected specimens for at least two months. In the second assay, where only the IP route was tested, the spleens and hearts of infected fish displayed higher viral amounts than the other organs tested. This suggests preferential tropism of the virus for these two organs, which could be compared (at least for the heart) with PMCV [39,40], even if the mid-kidney also appears to be involved in the early amplification of the virus before it is transferred to the target organ, the heart [13].

The immune response following toti-like virus infection in fish has not yet been described in detail in the literature. Even with the more described PMCV infection, only immune gene expression has been reported through transcriptomic studies, which provide evidence of early IFN-dependent gene activation, followed by B cell and MHC pathway activation [40]. Here, the humoral immune response was evaluated using ELISA performed on sera from infected fish sampled from 1 to 35 dpi (second experimental trial). The results provide evidence of the development of a humoral-specific response in experimentally infected sea bass as early as at 7 dpi for sea bass fingerlings infected intraperitoneally and that is sustained over time (high number of seropositive samples at 73 days and 4 months dpi). Several samples collected directly from the hatchery at different times also revealed the presence of various levels of specific anti-SBTLV antibodies.

Thus, even if no clinical signs are observed in infected fingerlings or mature fish, SBTLV triggers an adaptive immune response, as demonstrated by the production of specific antibodies. The apparent stable amount of virus in the internal organs until 35 dpi, a period during which the humoral response has developed, suggests that this specific response is not efficient enough to control the viral load. Other specific immune mechanisms, particularly the cytotoxic T lymphocyte response mediated by the MHC, may be involved in viral control, as demonstrated for PMCV infection in salmon, for which upregulation of complement-associated genes was observed immediately before an upregulation of the genes involved in the T-cell response [34].

Some gonad samples from mature sea bass exhibited positive SBTLV signals following a purification and concentration protocol. This result, with the absence of virus detection in internal organs from the same genitors, could suggest potential vertical transmission, even if strict analysis of sperm and eggs instead of gonads would give more insight into this hypothesis. A recent study focusing on the vertical transmission of PMCV, showed that the virus was detected in all the studied stages (eggs, larvae, fingerlings, and pre-smolt salmon) of progeny from PMCV-positive genitors [41]. Nevertheless, no clinical signs of CMS were observed in hatcheries or in later intermediate stages, such as juveniles or pre-smolts. Mikalsen et al. [42] failed to detect viral particles after reproduction by PMCV-positive genitors and artificial fertilization of salmon eggs [42]. This could be consistent with the maintenance of the virus at very low levels (below LOD) in the very early stages of development and the inefficacy of the immune response at these stages to clear the virus, opening the way to its persistence until the adult stage.

This study describes the isolation and characterization of a novel toti-like virus from sea bass larvae, SBTLV. Even if the virus was regularly and widely detected between 2018 and 2022 in the hatchery, inducing the development of sustainable specific humoral responses, the pathogenicity of this virus remains to be elucidated. The very narrow time window when the virus can be detected (larvae) makes it difficult to assess its pathogenicity through in vivo trials. Our experimental trials were performed with the SBTLV strain QQ40, which was isolated after long-term culture of BF2 cells. This strain was pure; however, the initial sample from the larvae contained a mix of two strains (RR200-MINOR and RR200-MAJOR). These strains differed by 147 mutations, among which one resulted in a potentially extended ORF2, which seems highly unlikely if RdRp is produced as a fusion protein with the capsid. Another mutation results in the loss of a potential ribosome slippery site at the 3′ end of ORF2. If this slippery site plays a functional role during the infection process, the possibility that the pathogenicity is linked either to the mix of strains or to the minor strain cannot be excluded. Recently, another toti-like virus, with a high level of similarity to the minor variant we describe (98.8% nt identity), was detected in another sea bass hatchery after a serious mortality event; once again this was in the early larval stages. This observation suggests that SBTLV is present in the production environment and could, depending on the circumstances and potential co-factors, become a health and economic burden in the same way PMCV has.

## Figures and Tables

**Table 1:** List and characteristics of the samples analyzed. Samples were obtained at different dates between February 2018 and November 2019 from healthy or dying sea bass at different ages (dph). They were analyzed by either cell culture performed with different cell lines at 14 °C or conventional RT-PCR specifically designed to detect the toti-like virus. * Consensus full-genome sequences were obtained by NGS for two different samples. CPEs: cytopathic effects; POS: positive; NEG: negative; np: not performed.

Sampling Date	Internal ID	Age (in dph)	Status	Cell Culture Analysis	NGS Analysis	Conventional RT-PCR
5 February 2018	QQ40b *	27	Dying	CPEs on BF2, LEB, and WSSK1 at 14 °C	POS	np
5 February 2018	QQ41	27	Dying	CPEs on BF2, LEB, and WSSK1 at 14 °C	np	POS
5 February 2018	QQ42	22	Dying	CPEs on BF2 (week signal)	np	POS
7 March 2019	QQ111	33	Dying	CPEs on BF2, LEB, and WSSK1 at 14 °C	np	POS
7 March 2019	QQ112	30	Healthy	CPEs on BF2, LEB, and WSSK1 at 14 °C	np	POS
23 April 2018	QQ118	21	Healthy	CPEs on BF2 (week signal)	np	POS
23 April 2018	QQ119	16	Healthy	NEG	np	NEG
12 November 2019	SS200	31	Dying	CPEs on LEB cells	np	np
13 November 2019	SS52	32	Dying	CPEs on LEB cells	np	np
12 November 2019	RR200 *	31	Dying	CPEs on LEB cells	POS	np

**Table 2:** Primers and probes used for detection and sequencing of the toti-like virus. The MS2 bacteriophage was used as a process control for the extraction and amplification process.

Identification	Sequence	Target	Position	%GC	Length	Tm	PCR Product Size (bp)
RT-qPCR (detection)							
oPVP446	Forward: CTCTGAGAGCGGCTCTATTGGT	MS2 bacteriophage	Replicase gene	54	22	66	101
oPVP447	Reverse: GTTCCCTACAACGAGCCTAAATTC			46	24	64	
tqPVP25	FAM-TCAGACACGCGGTCCGCTATAACGA-BHQ1			56	25	75	
oPVP559	Forward: CCGAGGCTATCAAAGTCAGC	Toti-like virus	3917–3936	55	20	60	110
oPVP560	Reverse: GCAGTCCATCTCCAACCACT		4007–4026	55	20	60	
tqPVP33	FAM-AGGATCGTTTCTGGCGTCAAAGAAT-BHQ1			44	25	70.2	
Conventional RT-PCR (detection and sequencing)							
oPVP592	Forward: CGCCATCTGGATTTGCAGAC	Toti-like virus	81 to 101	55	20	60	888
oPVP593	Reverse: CAGGATGAGGAACGCTGGTT		948 to 968	55	20	60	
oPVP594	Forward: ACAGCGTCGGAACAGACATT		926 to 946	50	20	60	944
oPVP595	Reverse: CTCCGCTCGCATCTGGTTAT		1849 to 1869	55	20	60	
oPVP596	Forward: AGTGCGTCTGAACAGAGTGG		1740 to 1760	55	20	60	845
oPVP597	Reverse: CTCGCCCTTGCCTGATGTTA		2564 to 2584	55	20	60	
oPVP532	Forward: AATGGAGTGGGTTGAACTCG		2353 to 2373	50	20	60	983
oPVP533	Reverse: TCATCAACCATTTCCTCGTG		3317 to 3336	45	20	60	
oPVP598	Forward: AAGGGTTCGGCGATCATTGG		3250 to 3270	55	20	61	851
oPVP599	Reverse: AACTATTCCCCTTGCGACCG		4080 to 4100	55	20	60	
oPVP600	Forward: TTCGGGCATCAGGAGCATTT		4060 to 4080	50	20	60	860
oPVP601	Reverse: TGTTGAAATCCAGTTCCCCGT		4899 to 4919	48	20	60	
oPVP602	Forward: TTGGACACGGGTTGAGGATC		4804 to 4824	55	20	60	821
oPVP603	Reverse: CCCTGTTCCCATGTGCTTCT		5604 to 5624	55	20	60	
oPVP604	Forward: TGCGTACTGAGACCACCAAG		5585 to 5605	55	20	60	812
oPVP605	Reverse: AAGCGATAACGTCAGGTGCA		6376 to 6396	50	20	60	
oPVP606	Forward: TGCACCTGACGTTATCGCTT		6377 to 6397	50	20	60	269
oPVP607	Reverse: GTGTGTCTTCGTCCTCTTCGT		6625 to 6645	52	20	60	
oPVP610	Forward: ACCGGAGAGCTTGGTTTTCG		6273 to 6293	55	20	64	211
oPVP611	Reverse: TTCCCCTCCATCTCCAACCA		6474 to 6494	55	20	65	
oPVP612	TTGCAAGCCCTTTCCTTCCA	primer 1 5′RACE PCR	359 to 379	50	20	65	257
oPVP615	ATGTAGAGTAGCAGGTCTGTCAAACCC	primer 2 5′RACE PCR	275 to 301	48	27	67	
oPVP616	TAGAAGTAGCTCATGGTTAGGACC	primer 3 5′RACE PCR	232 to 255	46	24	61	
oPVP629	AGGTCCGATGAGCGTATTGAAACTAC	primer 3′RACE	6504	46	26	68	315
Positive control							
oPVP608	Forward +T7: ATTGTTAATACGACTCACTATAGGGAACAGGAGGGGTGTGTTTGT	In vitro RNA	3804 to 3823	42	45	78	286
oPVP609	Reverse: CCCGAAGGATCGATAAGTTAA		4045 to 4065	43	21	62	

**Table 3:** List of Genbank accession numbers, Bioprojects, Biosamples, and SRA for the four submitted sequences (SeqId).

Accession Number	SeqId	Bioproject	Biosample	SRA (SRR Accession)
OQ791576	Toti-like_virus_isolate_QQ40-1stP-BF2	PRJNA930349	SAMN33713179	SRR24070899
OQ791577	Toti-like_virus_RR200-MINOR	PRJNA930359	SAMN33713174	SRR24072066
OQ791578	Toti-like_virus_RR200-MAJOR	PRJNA930359	SAMN33713175	SRR24072066
OQ791581	Toti-like_virus_isolate_QQ40-10thP-LEB	PRJNA930356	SAMN33713178	SRR24070344

**Table 4:** RT-qPCR detection of the toti-like virus in various organs from infected fingerlings (first experimental assay). The numbers correspond to the Ct obtained in RT-qPCR. Codification: B1 to B28: various organs sampled from fish infected by immersion; I1 to I28: organs sampled from fish infected by immersion; T1 to T7: negative control fish sampled at 16 dpi. nd: not detected; -: not performed.

Mode of Contamination	Organs	Days Post-Infection							
		16		23		30		60	
Bath	blood	B1	nd	B8	nd	B15	-	B22	nd
	brain	B2	nd	B9	nd	B16	-	B23	nd
	kidney	B3	nd	B10	nd	B17	nd	B24	nd
	spleen	B4	31.48	B11	nd	B18	nd	B25	nd
	heart	B5	nd	B12	nd	B19	nd	B26	nd
	dorsal fin	B6	nd	B13	35.99	B20	nd	B27	nd
	gills	B7	35.04	B14	nd	B21	nd	B28	nd
IP injection	blood	I1	37.73	I8	nd	I15	-	I22	nd
	brain	I2	nd	I9	nd	I16	nd	I23	nd
	kidney	I3	31.93	I10	32.34	I17	32.46	I24	34.11
	spleen	I4	29.93	I11	28.35	I18	28.70	I25	31.53
	heart	I5	30.04	I12	29.56	I19	30.27	I26	31.01
	dorsal fin	I6	34.51	I13	35.70	I20	29.67	I27	nd
	gills	I7	34.91	I14	35.07	I21	35.95	I28	nd
Negative control	blood	T1	nd						
	brain	T2	nd						
	kidney	T3	nd						
	spleen	T4	nd						
	heart	T5	nd						
	dorsal fin	T6	nd						
	gills	T7	nd						

**Table 5:** Screening of gonad samples received from the hatchery. a: RT-qPCR analysis on sea bass (*n* = 10) and seabream (*n* = 5) samples after direct nucleic acid extraction or after a purification/concentration step. Ct values and viral amount/PCR reaction are indicated for each sample after purification step. nd = not detected. b: Gonads from fourteen sea bass batches were purified/concentrated through an ultracentrifuge step with a 28% sucrose cushion and analyzed via RT-qPCR. Ct values and viral amount.reaction^−1^ are indicated for each sample. nd = not detected.

**a**				
**Label**	**Tank**	**Species**	**Concentration by Ultracentrifuge**	
			**Ct**	**Amount (cp.Reaction^−1^)**
1	A	BASS	nd	-
2			26.89	2.85 × 10^6^
3			35.21	7.93 × 10^3^
4			32.56	5.18 × 10^4^
5			33.72	2.27 × 10^4^
1	B	BASS	35.98	2.69 × 10^3^
2			nd	-
3			35.28	7.53 × 10^3^
4			nd (limit of detection)	-
5			32.71	4.66 × 10^4^
1	C	BREAM	32.57	2.84 × 10^4^
2			33.32	3.03 × 10^4^
3			nd	-
4			nd (limit of detection)	-
5			39.57 (below LOD)	3.64 × 10^2^
**b**				
**Sample**	**Gonads**			
	**Ct**		**Amount.Reaction^−1^**	
1A	31.47		1.42 × 10^5^	
1B	31.93		9.67 × 10^4^	
1C	36.05		3.07 × 10^3^	
1D	36.37		2.34 × 10^3^	
2A	35.41		6.38 × 10^3^	
2B	nd		nd	
2C	nd		nd	
2D	36.77		1.68 × 10^3^	
2E	37.44		1.21 × 10^3^	
3A	39.26		2.09 × 10^2^	
3B	32.04		8.82 × 10^4^	
3C	34.55		1.08 × 10^4^	
3D	37.20		1.17 × 10^3^	
3E	34.58		1.25 × 10^4^	

## Data Availability

The sequences presented in this study are openly available in GenBank under the SRR accessions SRR24070899, SRR24072066, SRR24072066, and SRR24070344. Complementary data are available in Supplementary Materials (Table S2).

## Supplementary Materials

The following supporting information can be downloaded at: https://www.mdpi.com/article/10.3390/v15122423/s1. Table S1: Additional samples of sea bass or seabream larvae, sea bass eggs, Artemia, or isolated internal sea bass organs, analyzed at various times from 2018 to the end of 2019, with healthy or dying status, to obtain an epidemiological overview. Ranges of age were identified as “A” for larvae from 5 to 20 dph, “B” between 21 and 30 dph, and “C” older than 31 dph. Samples were all analyzed using RT-qPCR targeting the putative toti-like virus; Table S2: Screening of supplementary and diversified samples coming from the hatchery. A total of 72 samples from sea bass larvae aged from 5 days to 53 days, 6 from isolated internal organs (79 dph larvae), 2 from eggs, 4 from seabream larvae and 2 from Artemia were analyzed via RT-qPCR. Health status (dying or healthy), as well as age class (A for larvae before 20 dph, B for between 21 and 30 dph, and C after 31 dph) are specified for larvae samples. Ct values and viral amount·mg^−1^ in tissue are indicated for each sample. nd = not detected.
