# Supplementary material for: Characterization of a New Toti-like Virus in Sea Bass, Dicentrarchus labrax
Source: Viruses. 2023 Dec 13;15(12):2423. doi: 10.3390/v15122423 (PMC10748352; doi:10.3390/v15122423)
Supplement: Supplementary file 1 [file viruses-15-02423-s001.zip › viruses-2768481-supplementary.pdf]

Table S1. Additional samples of sea bass or seabream larvae, sea bass eggs, artemia, or isolated internal sea bass organs, analyzed at various time from 2018 to the end of 2019, with healthy or dying status, to get an epidemiological overview. Ranges of age were identified as "A" for larvae from 5 to 20 dph, "B" between 21 and 30 dph, "C" older than 31 dph. Samples were all analyzed by RT-qPCR targeting the putative Toti-like virus.

Samples	Batch	Number of days post-hatchering (dph)	Age class	Health status
sea bass larvae	1	5	A	Healthy
		10	A	Healthy
		15	A	Healthy
		20	A	Healthy
		20	A	Healthy
		22	B	Dying
		22	B	Healthy
		25	B	Dying
		25	B	Healthy
		27	B	Dying
		27	B	Healthy
		29	B	Dying
		29	B	Healthy
		30	B	Healthy
		30	B	Healthy
		31	C	Dying
		31	C	Healthy
		33	C	Healthy
		36	C	Healthy
	2	20	A	Healthy
		22	B	Healthy
		22	B	Healthy
		25	B	Healthy
		25	B	Healthy
		27	B	Dying
		27	B	Healthy
		27	B	Healthy
		29	B	Dying
		29	B	Dying
		29	B	Healthy
		29	B	Healthy
		30	B	Healthy
		30	B	Healthy
		30	B	Dying
		31	C	Healthy
		31	C	Healthy
		31	C	Healthy
	3	24	B	Dying
		26	B	Dying
	4	31	C	Healthy
	5	32	C	Dying

		32	C	Dying
		32	C	Dying
		32	C	Dying
		32	C	Healthy
		32	C	Healthy
		32	C	Healthy
		33	C	Dying
		33	C	Dying
		33	C	Dying
		33	C	Dying
		33	C	Healthy
		33	C	Healthy
		33	C	Healthy
		33	C	Healthy
		33	C	Healthy
		28-31	B-C	Healthy
	6	23	C	Healthy
		23	B	Healthy
		24	B	Healthy
		24	B	Healthy
		31	C	Dying
		32	C	Healthy
		32	C	Healthy
		33	C	Healthy
		33	C	Healthy
	7	53	C	Healthy
	8	43	C	Healthy
	9	40	C	Healthy
	10	38	C	Healthy
	11	38	C	Healthy
	12	53	C	Healthy
sea bass organs	heart	79	C	Healthy
	spleen	79	C	Healthy
	kidney	79	C	Healthy
	heart	79	C	Healthy
	spleen	79	C	Healthy
	kidney	79	C	Healthy
sea bass eggs	-	-	-	Healthy
	-	-	-	Healthy
Artemia	-	-	-	-
	-	-	-	-
seabream larvae	-	5	A	Healthy
	-	5	A	Healthy
	-	5	A	Healthy
	-	5	A	Healthy

Table S2. Screening of supplementary and diversified samples coming from the hatchery. A total of 72 samples from sea bass larvae aged of 5 days to 53 days, six

of isolated internal organs (79 dph larvae), 2 of eggs, 4 of seabream larvae and 2 of artemia were analyzed by RT-qPCR. Health status (dying or healthy) as well as age class (A for larvae before 20DPH, B between 21 and 30, and C after 31 dph) are specified for larvae samples. Ct values and viral amount.mg^{-1} of tissue are indicated for each sample. nd = not detected.

Sample Id	Kind of samples	Age class	Number of days post-hatchering (dph)	Health status	Ct	Number of copies.mg^{-1} of tissue
1		B	22	Dying	34.4	3.00E+04
2		B	24	Dying	27.83	1.67E+06
3		B	25	Dying	20.55	1.44E+08
4		B	26	Dying	23.41	2.50E+07
5		B	27	Dying	21.31	9.04E+07
6		B	29	Dying	20.98	1.10E+08
7		B	30	Dying	19.34	3.02E+08
8		C	31	Dying	18.32	5.60E+08
9		B	27	Dying	17.647	5.90E+08
10		B	29	Dying	16.675	1.06E+09
11		B	29	Dying	22.637	2.90E+07
12		C	32	Dying	17.49	1.43E+09
13		C	32	Dying	17.17	1.79E+09
14		C	33	Dying	19.65	3.16E+08
15		C	33	Dying	20.67	1.55E+08
16		C	32	Dying	18.45	7.34E+08
17		C	33	Dying	17.1	1.89E+09
18		C	32	Dying	19.28	4.12E+08
19		C	33	Dying	19.48	3.56E+08
20		C	31	Dying	18.96	
21	sea bass larvae	A	5	Healthy	nd	0.00E+00
22		A	10	Healthy	nd	0.00E+00
23		A	15	Healthy	nd	0.00E+00
24		A	20	Healthy	30.86	2.62E+05
25		B	22	Healthy	33.39	5.56E+04
26		B	25	Healthy	21.54	7.82E+07
27		B	27	Healthy	20.32	1.65E+08
28		B	29	Healthy	23.7	2.08E+07
29		B	30	Healthy	26.75	3.24E+06
30		C	31	Healthy	22.5	4.36E+07
31		C	33	Healthy	26.49	3.78E+06
32		C	36	Healthy	24.69	1.14E+07
33		A	20	Healthy	nd	0.00E+00
34		A	20	Healthy	nd	0.00E+00
35		B	22	Healthy	33.924	3.17E+04
36		B	22	Healthy	32.066	9.74E+04
37		B	25	Healthy	24.963	7.11E+06
38		B	25	Healthy	21.176	7.00E+07
39		B	27	Healthy	20.233	1.24E+08
40		B	27	Healthy	18.747	3.04E+08
41		B	29	Healthy	20.798	8.80E+07
42		B	29	Healthy	23.264	1.98E+07

43		B	30	Healthy	19.105	2.45E+08
44		B	30	Healthy	19.518	1.91E+08
45		B	30	Healthy	23.947	1.31E+07
46		C	31	Healthy	20.377	1.13E+08
47		C	31	Healthy	26.161	3.45E+06
48		C	31	Healthy	22.032	4.18E+07
49			28-31	Healthy	17.2	1.75E+09
50		C	32	Healthy	20.47	1.78E+08
51		C	33	Healthy	19.53	3.44E+08
52		C	32	Healthy	22.43	4.52E+07
53		C	33	Healthy	21.72	7.42E+07
54		C	32	Healthy	19.71	3.04E+08
55		C	33	Healthy	18.82	5.66E+08
56		C	33	Healthy	22.9	3.26E+07
57		C	33	Healthy	20.79	1.43E+08
58		C	32	Healthy	24.85	8.36E+06
59		C	33	Healthy	26.53	2.58E+06
60		C	32	Healthy	28.08	8.72E+05
61		C	33	Healthy	28.15	8.32E+05
62		B	23	Healthy	23.74	1.81E+07
63		B	24	Healthy	22.7	3.76E+07
64		B	23	Healthy	26.61	2.44E+06
65		B	24	Healthy	26.6	2.46E+06
66		C	31	Healthy	33.93	1.46E+04
67		C	53	Healthy	24.34	1.23E+07
68		C	43	Healthy	24.27	1.29E+07
69		C	40	Healthy	25.37	6.13E+06
70		C	38	Healthy	23.15	2.73E+07
71		C	38	Healthy	19.75	2.67E+08
72		C	53	Healthy	26.87	2.25E+06
73	sea bass organs	-	79	Heart	33.71	2.28E+04
74		-	79	Spleen	36.25	4.12E+03
75		-	79	Kidney	35.11	8.85E+03
76		-	79	Heart	34.22	1.61E+04
77		-	79	Spleen	29.88	2.98E+05
78		-	79	Kidney	33.15	3.31E+04
79	sea bass eggs	-	-	Healthy	nd	0.00E+00
80		-	-	Healthy	nd	0.00E+00
81	Artemia	-	-	-	39.29	1.51E+03
82		-	-	-	nd	0.00E+00
83	seabream larvae	-	5	Healthy	nd	0.00E+00
84		-	5	Healthy	nd	0.00E+00
85		-	5	Healthy	nd	0.00E+00
86		-	5	Healthy	nd	0.00E+00

Sample Id	Kind of samples		Number of days post-hatchering (dph)	Health status	Ct	Number of copies.mg^{-1} of tissue
1	sea bass larvae	B	22	Dying	34.4	3.00E+04
2		B	24	Dying	27.83	1.67E+06
3		B	25	Dying	20.55	1.44E+08
4		B	26	Dying	23.41	2.50E+07
5		B	27	Dying	21.31	9.04E+07
6		B	29	Dying	20.98	1.10E+08
7		B	30	Dying	19.34	3.02E+08
8		C	31	Dying	18.32	5.60E+08
9		B	27	Dying	17.647	5.90E+08
10		B	29	Dying	16.675	1.06E+09
11		B	29	Dying	22.637	2.90E+07
12		C	32	Dying	17.49	1.43E+09
13		C	32	Dying	17.17	1.79E+09
14		C	33	Dying	19.65	3.16E+08
15		C	33	Dying	20.67	1.55E+08
16		C	32	Dying	18.45	7.34E+08
17		C	33	Dying	17.1	1.89E+09
18		C	32	Dying	19.28	4.12E+08
19		C	33	Dying	19.48	3.56E+08
20		C	31	Dying	18.96	
21		A	5	Healthy	nd	0.00E+00
22		A	10	Healthy	nd	0.00E+00
23		A	15	Healthy	nd	0.00E+00
24		A	20	Healthy	30.86	2.62E+05
25		B	22	Healthy	33.39	5.56E+04
26		B	25	Healthy	21.54	7.82E+07
27		B	27	Healthy	20.32	1.65E+08
28		B	29	Healthy	23.7	2.08E+07
29		B	30	Healthy	26.75	3.24E+06
30		C	31	Healthy	22.5	4.36E+07
31		C	33	Healthy	26.49	3.78E+06
32		C	36	Healthy	24.69	1.14E+07
33		A	20	Healthy	nd	0.00E+00
34		A	20	Healthy	nd	0.00E+00
35		B	22	Healthy	33.924	3.17E+04
36		B	22	Healthy	32.066	9.74E+04
37		B	25	Healthy	24.963	7.11E+06
38		B	25	Healthy	21.176	7.00E+07
39		B	27	Healthy	20.233	1.24E+08
40		B	27	Healthy	18.747	3.04E+08
41		B	29	Healthy	20.798	8.80E+07
42		B	29	Healthy	23.264	1.98E+07
43		B	30	Healthy	19.105	2.45E+08
44		B	30	Healthy	19.518	1.91E+08
45		B	30	Healthy	23.947	1.31E+07

46		C	31	Healthy	20.377	1.13E+08
47		C	31	Healthy	26.161	3.45E+06
48		C	31	Healthy	22.032	4.18E+07
49			28-31	Healthy	17.2	1.75E+09
50		C	32	Healthy	20.47	1.78E+08
51		C	33	Healthy	19.53	3.44E+08
52		C	32	Healthy	22.43	4.52E+07
53		C	33	Healthy	21.72	7.42E+07
54		C	32	Healthy	19.71	3.04E+08
55		C	33	Healthy	18.82	5.66E+08
56		C	33	Healthy	22.9	3.26E+07
57		C	33	Healthy	20.79	1.43E+08
58		C	32	Healthy	24.85	8.36E+06
59		C	33	Healthy	26.53	2.58E+06
60		C	32	Healthy	28.08	8.72E+05
61		C	33	Healthy	28.15	8.32E+05
62		B	23	Healthy	23.74	1.81E+07
63		B	24	Healthy	22.7	3.76E+07
64		B	23	Healthy	26.61	2.44E+06
65		B	24	Healthy	26.6	2.46E+06
66		C	31	Healthy	33.93	1.46E+04
67		C	53	Healthy	24.34	1.23E+07
68		C	43	Healthy	24.27	1.29E+07
69		C	40	Healthy	25.37	6.13E+06
70		C	38	Healthy	23.15	2.73E+07
71		C	38	Healthy	19.75	2.67E+08
72		C	53	Healthy	26.87	2.25E+06
73	sea bass organs	-	79	Heart	33.71	2.28E+04
74		-	79	Spleen	36.25	4.12E+03
75		-	79	Kidney	35.11	8.85E+03
76		-	79	Heart	34.22	1.61E+04
77		-	79	Spleen	29.88	2.98E+05
78		-	79	Kidney	33.15	3.31E+04
79	sea bass eggs	-	-	Healthy	nd	0.00E+00
80		-	-	Healthy	nd	0.00E+00
81	Artemia	-	-	-	39.29	1.51E+03
82		-	-	-	nd	0.00E+00
83	seabream larvae	-	5	Healthy	nd	0.00E+00
84		-	5	Healthy	nd	0.00E+00
85		-	5	Healthy	nd	0.00E+00
86		-	5	Healthy	nd	0.00E+00
